# Realization of High-Power Single-Frequency Continuous-Wave Tunable 689 nm Laser

**DOI:** 10.3390/mi17020200

**Published:** 2026-02-01

**Authors:** Jiao Wei, Jingru Qiao, Pixian Jin, Jing Su, Huadong Lu

**Affiliations:** 1 State Key Laboratory of Quantum Optics Technologies and Devices, Shanxi University, Taiyuan 030006, China; weijiao@sxu.edu.cn (J.W.); 13835720392@163.com (J.Q.); pxjin@sxu.edu.cn (P.J.); jingsu@sxu.edu.cn (J.S.); 2 Collaborative Innovation Center of Extreme Optics, Shanxi University, Taiyuan 030006, China

**Keywords:** high-power, single-frequency, thermal effect, strontium atoms

## Abstract

By analyzing the influence of the titanium–sapphire (Ti:S) crystal thermal effect on the laser resonator during the generation of a 689 nm laser, the thermal characteristics of the Ti:S crystal operating near the gain edge were investigated in this letter. On this basis, a Ti:S laser with high conversion efficiency suitable for operation at the wavelength of 689 nm was designed. Benefiting from the quantification of thermal effects, the beam waist size at the center of the Ti:S crystal was precisely controlled. Finally, a single-frequency continuous-wave 689 nm laser with an output power of 3.65 W was achieved, and the corresponding optical-to-optical conversion efficiency was up to 23.1%. Then, after locking the transmission peak of the inserted etalon to the resonance frequency of the resonator, the continuous-frequency tuning range of 17 GHz around 689 nm was realized by scanning the voltage applied to the piezoelectric transducer (PZT) mounted on the cavity mirror. Furthermore, based on the realized single-frequency continuous-wave tunable 689 nm laser source, the absorption spectra of strontium atoms near 689 nm were obtained, which established a promising method for preparing 689 nm laser sources designed for strontium atomic ensembles.

## 1. Introduction

Strontium atoms [1] have attracted a lot of attention in precision measurement [2], quantum computing [3], and quantum simulation [4] owing to their excellent properties of ultranarrow-linewidth transition and insensitivity to both thermal effects and stray electromagnetic fields [5]. In the field of precision measurement, optical clocks based on strontium atoms operated at a frequency (∼$4.3\times 10^{14}$ Hz) that is about five orders of magnitude higher than that of traditional microwave clocks based on cesium atoms (∼$9.2\times 10^{9}$ Hz) [6]. Theoretically, this made strontium atomic clocks superior to cesium atomic clocks in terms of stability and lower uncertainty [7]. In 2024, A. Aeppli et al. experimentally validated that the frequency uncertainty of the strontium clock systems had reached the $10^{-19}$ level [8], which surpassed the precision of the current definition of the second. In this field, the preparation of ultracold strontium atoms necessitates the use of an extremely narrow-linewidth 689 nm laser as the cooling light source. In the field of quantum simulation and quantum computation, quantum simulation platforms based on strontium atoms enable the emulation of a wide range of strongly correlated many-body quantum systems. Meanwhile, the high-power, narrow-linewidth, single-frequency 689 nm laser is the crucial factor for the preparation of optical lattices with larger spatial scales and longer coherence lifetimes. Therefore, it was important for these mentioned applications to prepare high-power, single-frequency, narrow-linewidth tunable 689 nm lasers.

As early as the 1990s, tunable dye lasers were the only option for generating tunable single-frequency 689 nm lasers [9]. However, dye lasers suffered from significant fluctuations in output power and frequency caused by the degradation of the dye solution after a period of usage, which made it difficult to meet the demands of long-term stable experiments. With the rapid development of semiconductor materials in the 20th century, it was found that the bandgap energy of AlGaInP materials precisely matched the conditions for emitting a 689 nm laser [10] and then it became one of the mainstream methods for generating a 689 nm laser. However, the physical properties of AlGaInP materials, such as the valence band structure and doping challenges, directly led to a high threshold current, low efficiency, and high thermal sensitivity. One of the most representative examples was the DL-Pro 689 nm external cavity diode laser (ECDL). Even with a cascaded tapered amplifier, it could only achieve an output power of 100 mW. Many research groups had successfully obtained ultracold strontium atoms using frequency-stabilized ECDL [11]. The emergence of Discrete Mode Lasers (DMLs) effectively overcame this problem. However, the output power was only 10 mW [12]. Additionally, P. H. Moriya et.al reported an AlGaInP-based vertical external cavity surface-emitting laser (VECSEL) pumped by a high-power blue InGaN diode laser and achieved 140 mW of a single-frequency continuous-wave 689 nm laser. The linewidth of the achieved 689 nm laser was narrowed to the sub-kHz level by locking it to a reference cavity [13]. Besides directly generating a 689 nm red laser, researchers had also explored sum-frequency generation based on mature infrared semiconductor technology. In 2011, X. M. Fan et al. successfully generated 156 mW of continuous-wave 689 nm light by sum-frequency mixing 18.2 W of a 1444 nm laser with 12.7 W of a 1319 nm laser [14]. Overall, the output power of 689 nm lasers remained at the hundred-milliwatt level. It was evident that the available power level was insufficient to meet the demanding requirements for constructing large-scale optical lattices in quantum simulations using a 689 nm laser [15]. By comparison, it was a potential method to generate a 689 nm laser by employing Ti:Sapphire (Ti:S) crystal with an ultra-broad gain spectrum as the gain medium [16]. The Matisse CR series Ti:S laser from Spectra-Physics (UK) had generated 4.4 W of a 689 nm laser under 25 W pumping and the optical-to-optical conversion efficiency was 17.6%. However, the low efficiency caused by the low gain of 689 nm at the edge of the Ti:S gain spectrum [17] inevitably led to severe thermal load on the Ti:S crystal, thereby affecting the power stability of the Ti:S laser, which would directly impact the cooling rate of cold strontium atoms [18]. Therefore, it was necessary to analyze the thermal characteristics and design the resonator when the Ti:S laser operated at the wavelength of 689 nm.

In this work, we conducted specialized research on the gain and thermal characteristics of the Ti:S laser operating at 689 nm. Based on this, by precisely controlling the cavity mode and pump mode, a single-frequency continuous-wave 689 nm laser with a maximal output power of 3.65 W was achieved under 15.8 W pumping, and the corresponding optical-to-optical conversion efficiency was up to 23.1%. With the etalon locked, a continuous-frequency tuning range of 17 GHz was realized. Using this single-frequency tunable laser source, absorption spectra of strontium atoms near 689 nm were obtained by passing the laser beam through a homemade strontium atomic vapor cell. This work lays a fundamental foundation for further narrowing the laser linewidth and developing narrow-linewidth laser sources that meet the requirements for the second-stage cooling of strontium atoms.

## 2. Principle

**Figure 1:**
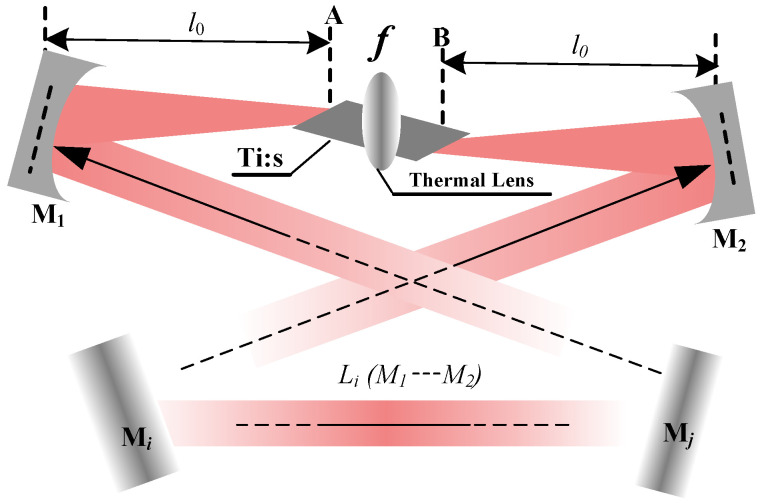
Schematic of a Ti:S ring resonator with thermal lensing effect.

To eliminate the spatial hole-burning effect, the four-mirror or multi-mirror ring resonator is typically adopted, as depicted in Figure 1. Meanwhile, owing to the inherently short fluorescence lifetime of the Ti:sapphire gain medium, the crystal must be precisely located at the intracavity beam waist between two concave mirrors ($M_{1}$ and $M_{2}$) to ensure a pump intensity high enough to initiate and sustain laser action. Operation at the gain edge, characterized by a small stimulated emission cross-section, inevitably induces pronounced thermal loading. Then, the thermal effects of the Ti:S crystal can be accurately modeled as a thermal lens with the focal length of *f*. In the resonator layout, A and B mark the front and rear facets of the Ti:S crystal, respectively, with the concave mirrors $M_{1}$ and $M_{2}$ positioned at a distance of $l_{0}$ from each facet. The remaining cavity mirrors ($M_{i}$… $M_{j}$) are all planar, defining an optical path length of $L_{i}$. Notably, the beam waist size within the crystal and the overall stability regime of the resonator exhibit minimal sensitivity to variations in $L_{i}$. Consequently, the present analysis is centered on the impact of the equivalent thermal lens focal length *f* and the distance $l_{0}$ on the operational characteristics of the resonator.

By employing the matrix optics formalism, an equivalent periodic thin-lens sequence that incorporates the thermal lens focal length as a key parameter can be derived by Equation (1) under the assumption that astigmatic aberrations are neglected.

(1) $$M=\left(\begin{array}{cc} a & b\\ c & d \end{array}\right)=\left(\begin{array}{cc} 1 & 2l_{0}+l_{Ti:s}\\ 0 & 1 \end{array}\right)\cdot \left(\begin{array}{cc} 1 & 0\\ -\frac{2}{r} & 1 \end{array}\right)\cdot \left(\begin{array}{cc} 1 & L_{i}\\ 0 & 1 \end{array}\right)\cdot \left(\begin{array}{cc} 1 & 0\\ -\frac{2}{r} & 1 \end{array}\right)\cdot \left(\begin{array}{cc} 1 & 0\\ -\frac{1}{f} & 1 \end{array}\right)$$

where $l_{Ti:s}$ is the length of the Ti:S crystal; r is the curvature radius of the cavity mirrors $M_{1}$ and $M_{2}$. The beam waist radius at the center of the Ti:S crystal can be calculated using the following Equation (2).

(2) $$w\left(l_{0},f\right)=\frac{\lambda }{2\pi c}\sqrt{4-{\left(a+d\right)}^{2}}$$

The stability range can be analyzed by the value of $s(l_{0},f)$, which is shown as follows.

(3) $$s(l_{0},f)=a+d$$

**Figure 2:**
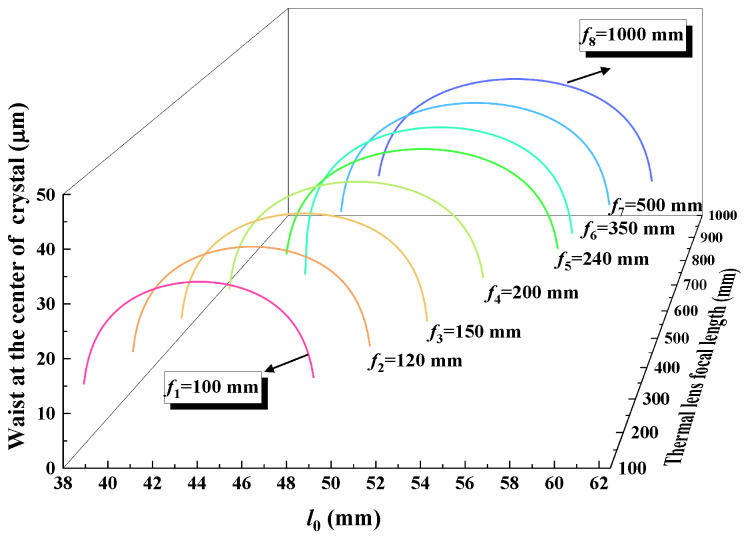
Relationship between the stability range of the resonator and the equivalent thermal focal length.

In order to analyze the impact of the thermal effect on the resonator, the variation in the stability range of a four-mirror ring resonator with the equivalent thermal focal length was theoretically calculated, which is shown in Figure 2. It was observed that the stability range of the resonator narrowed and moved to the left as the equivalent thermal focal length shortened, which verified that the change in the resonator caused by the thermal effect can be compensated by shortening the cavity length. When the Ti:S laser operated at the wavelength of 689 nm, the low gain led to a significant thermal effect. Based on the analysis above, we considered shortening the cavity length to compensate for the changes caused by the thermal effects. It was found that when the distances from the two concave mirrors $M_{1}$ and $M_{2}$ to the crystal facets $l_{0}$ was precisely controlled at 47.1 mm, the resonator achieved stable, efficient, and single-frequency operation. This implies that, at this specific cavity length, the thermal lensing effect of the Ti:S crystal is precisely compensated. Therefore, substituting $l_{0}$ = 47.1 mm into the Equation (2), we can calculate the value of *f*, which was 240 mm.

**Figure 3:**
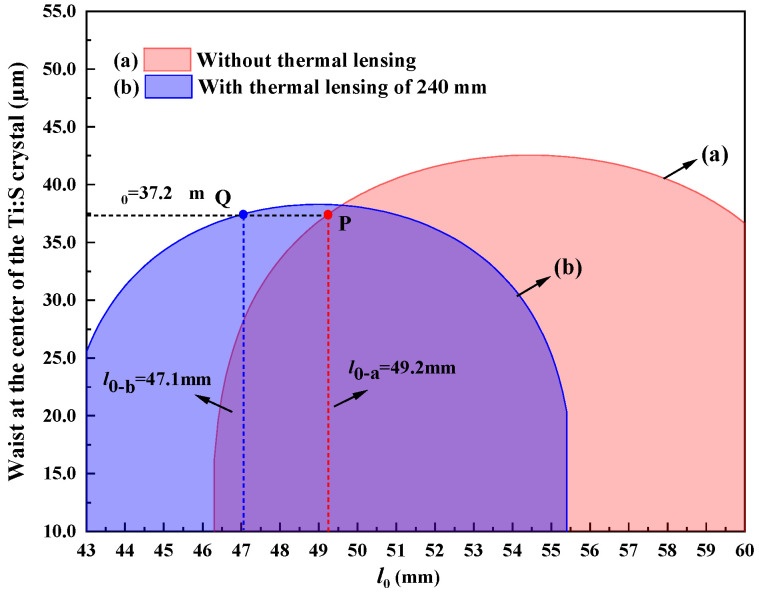
Waist at the center of the Ti:S crystal when the resonator was (**a**) without thermal lensing and (**b**) with thermal lensing of 240 mm.

Figure 3 clearly shows the variation in the waist at the center of the Ti:S crystal when the resonator was (a) without thermal lensing and (b) with thermal lensing of 240 mm. It was seen that $l_{0}$ needs to be adjusted from 49.2 mm to 47.1 mm when the resonator had thermal lensing of 240 mm. At point P and Q, the waist at the center of the Ti:S crystal can maintain the optimal size of 37.2 μm. It is theoretically demonstrated that the length $l_{0}$ of 47.1 mm provided perfect compensation for thermal effects. The conclusion proves that the stable operation of the laser is attributed to the precise design and control of the cavity length, and the heat generated by the low conversion efficiency in the small-gain regime of the Ti:S laser dominated over the positive effect of the small quantum defect when the Ti:S laser was operating at the wavelength of 689 nm.

**Figure 4:**
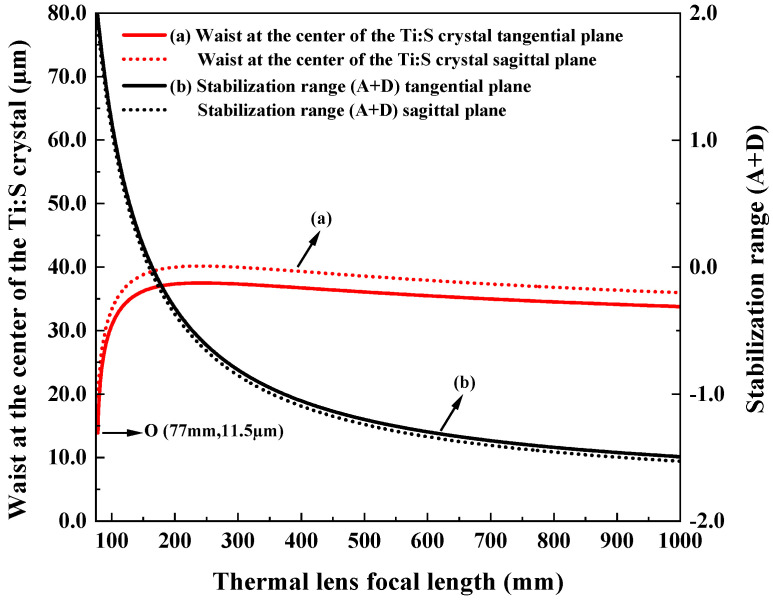
Variation in intracavity beam waist and stability range with the thermal lens focal length.

Then, the sensitivity of the designed resonator to the thermal effect was evaluated, and the results are shown in Figure 4. Obviously, for the thermal focal length above 200 mm, as the thermal focal length shortens, the beam waist became large. Once the thermal focal length was less than 200 mm, the beam waist decreased with the intensification of the thermal effect. Especially below 150 mm of the thermal focal length, the impact of the thermal effects on the resonator becomes more pronounced. It was clear that the resonator can tolerate a maximum equivalent thermal focal length of 77 mm (Point O).

## 3. Experiment

**Figure 5:**
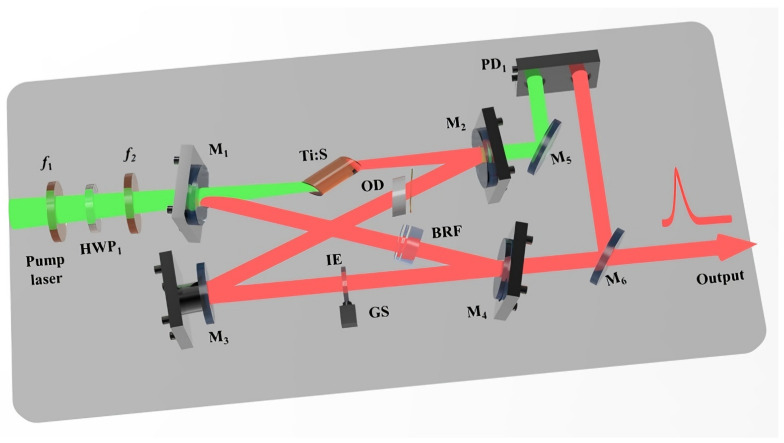
Schematic diagram of the designed high-power single-frequency continuous-wave tunable 689 nm laser.

The designed experimental setup is shown in Figure 5. The pump source was an all-solid-state single-frequency continuous-wave intracavity frequency-doubled 532 nm green laser (DPSS FG-VIIB, Yuguang Co., Ltd., Taiyuan, China) [19,20] with output power of 16 W. The pump laser first passed through a dichroic beam splitter to filter out the residual 1064 nm fundamental laser and then the purified 532 nm laser was focused onto the center of the Ti:S crystal by two plano-convex lenses $f_{1}$ and $f_{2}$ with a focal length of 200 mm and 110 mm, respectively. In order to compensate for the astigmatism introduced by the pump laser passing through the Brewster-cut Ti:S crystal, $f_{2}$ was positioned at a precisely adjusted tilt angle θ. The Ti:S crystal was with dimensions of ϕ4 × 20 mm. Considering the low quantum defect and low gain characteristics of the Ti:sapphire crystal when operating at 689 nm, the doping concentration was maintained at the classic choice of 0.05 wt.%. The resonator consisted of two concave mirrors ($M_{1}$ and $M_{2}$) and two plane mirrors ($M_{3}$ and $M_{4}$). Both concave mirrors had a radius of curvature of 100 mm. A piezoelectric transducer (PZT) with a length of 15 mm was attached to $M_{3}$ to enable continuous scanning of the cavity length. A birefringent filter (BRF) was used for coarse wavelength selection. To achieve laser oscillation at 689 nm, the coating of $M_{3}$ was specially designed (R > 99.5%@650–730 nm; R < 50%@750–1100 nm) to make the BRF operate at the interference order of *k* = 6. The optical diode, composed of a 2.5 mm thick TGG crystal and a 0.522 mm quartz plate, ensured unidirectional laser operation. In addition, an intracavity etalon with a thickness of 0.5 mm was used to lock its transmission peak to the resonance frequency of the resonator and then achieve stable single-frequency operation of the 689 nm laser.

## 4. Results and Discussion

**Figure 6:**
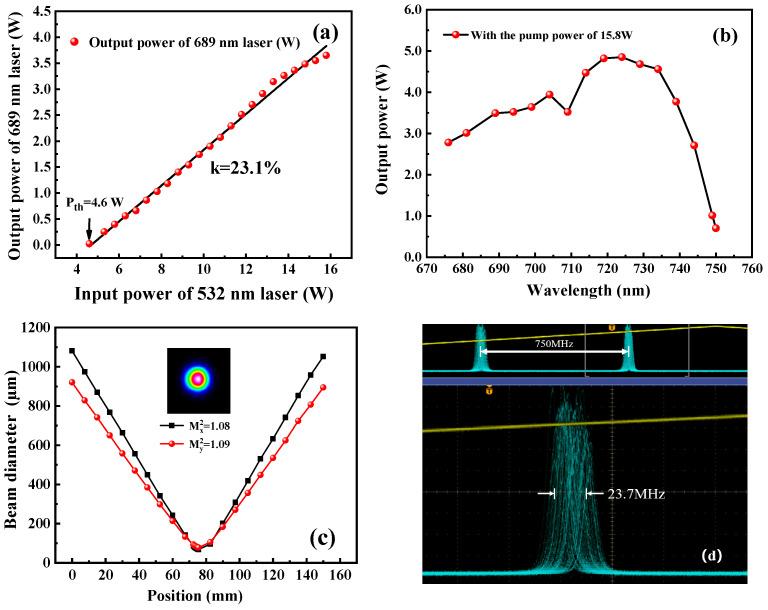
Output characteristics of the realized single-frequency continuous-wave 689 nm laser: (**a**) output power curve, (**b**) wavelength tuning curve, (**c**) beam quality, and (**d**) frequency drift.

The output power of the achieved 689 nm laser was monitored behind the output coupler $M_{6}$ using a power meter (PM100D, Thorlabs, Newton, NJ, USA). By gradually increasing the injected pump power, the variation in output power was recorded. As shown in Figure 6a, it was seen that the threshold pump power was 4.6 W. With the increasing of the injected pump power, the output power of the 689 nm laser was increased steadily. When the injected pump power reached 15.8 W, the maximum output power was up to 3.65 W. The overall optical-to-optical conversion efficiency was 23.1%. This value was comparable to the efficiency of the Ti:S laser operating at 795 nm [21,22,23,24,25]. Meanwhile, we focused on the power increase deviating slightly from the linear fitting curve, which was attributed to the dynamically varying thermal effect. The optimal mode matching between the pump laser mode and oscillating laser mode was achieved when the total pump power was injected. Then, by rotating the BRF, the output power at different wavelengths was monitored (AMOS-UVNIR, Lbtek, Shenzhen, China), and the tuning curve is shown in Figure 6b. It was seen that the wavelength could be tuned from 685 nm to 750 nm. The peak power of 4.7 W was observed at the wavelength of 725 nm. With the laser operating at 689 nm, the beam quality was measured by a beam quality analyzer (M2SETVIS, Thorlabs, Newton, NJ, USA). As shown in Figure 6c, the beam profile exhibited a Gaussian distribution, and the measured $M^{2}$ values in the X and Y directions were better than 1.08 and 1.09, respectively. It was also proved that our quantitative assessment of the equivalent thermal focal length of the Ti:S crystal was relatively accurate and the excellent mode matching between the pump beam and the designed intracavity oscillating beam was realized. When the transmission peak of the etalon was locked to the resonance frequency of the cavity, the laser achieved long-term stable single-frequency operation. The frequency drift of the 689 nm laser under free-running conditions over 10 s was recorded in Figure 6d, which was 23.7 MHz.

**Figure 7:**
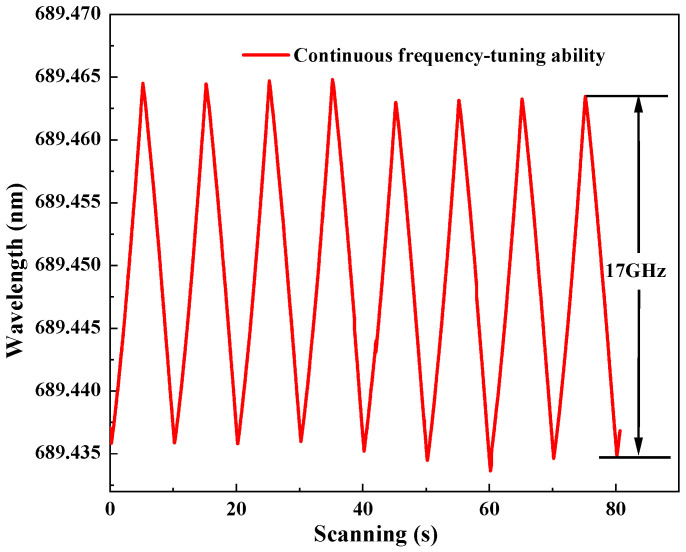
Continuous-frequency tuning characteristics of the single-frequency continuous-wave 689 nm laser.

Furthermore, the continuous-frequency tuning of the generated 689 nm laser was recorded (WS6-761, High Finesse Laser and Electronic Systems, Tübingen, Germany) as in Figure 7 when the voltage applied to the PZT on $M_{3}$ was continuously modulated by a driver with a frequency of 0.05 Hz. It was observed that the continuous-frequency tuning range reached 17 GHz.

**Figure 8:**
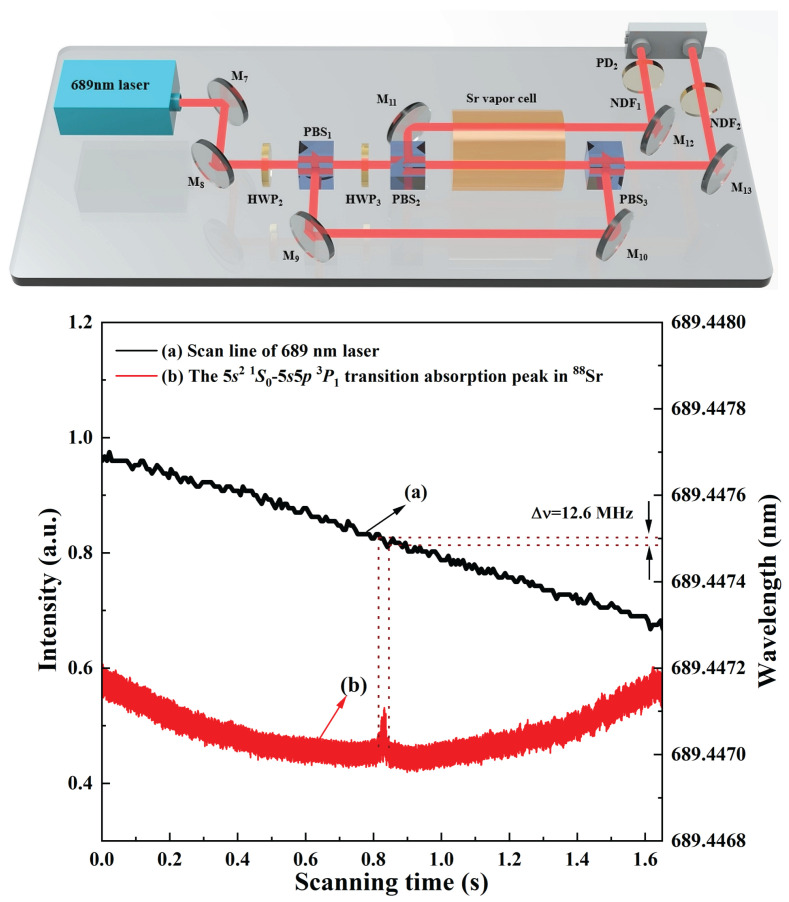
Strontium atomic saturated absorption spectroscopy setup and measured saturated absorption peak of ^88^Sr.

Based on this achieved 689 nm tunable laser source, a strontium atomic saturated absorption spectroscopy setup was constructed, which is shown in Figure 8. The strontium atomic vapor cell used in the experiment had a length of 50 cm and a clear aperture of 1.5 inches. To render the saturated absorption spectrum distinguishable from the background noise, the strontium vapor cell was heated to 610 °C using a nickel-chromium (NiCr) heating wire (305D II, ZHAO XIN PS, Shenzhen, China) wrapped around the tube wall. The strontium source was positioned at the center of the cell to ensure uniform vapor distribution. An outer layer of thermal insulation material was applied to the cell to maintain stable temperature conditions, which is critical for minimizing thermal fluctuations and ensuring consistent spectral measurements. The 689 nm laser beam was split by a half wave plate (${\mathrm{HWP}}_{2}$ and ${\mathrm{HWP}}_{3}$) and polarizing beam splitter (${\mathrm{PBS}}_{1}$ and ${\mathrm{PBS}}_{2}$) into a strong pump laser $I_{1}$, a weak probe laser $I_{2}$, and a reference laser $I_{3}$. $I_{1}$ and $I_{2}$ were aligned to counter-propagate through the strontium vapor-filled cell. The reference beam $I_{3}$ passes directly through the strontium vapor cell.

Finally, the differential signal between $I_{1}$ and $I_{3}$ was detected using a free-space balanced photodetector ${\mathrm{PD}}_{2}$ (PDB210A/M, Thorlabs, Newton, NJ, USA). Figure 8 displays the recorded laser frequency variation curve and the saturated absorption spectrum when continuously adjusting the voltage applied to the PZT mounted on the cavity mirror $M_{3}$. Figure 8a shows the frequency scanning curve of the high-power single-frequency continuous-wave 689 nm laser, and Figure 8b is the obtained saturated absorption peak of ^88^Sr. This absorption peak corresponds to the transition 5s^2^ ^1^S_0_→5s5p ^3^P_1_. The laser frequency variation corresponding to the full width at half maximum of the absorption peak was measured to be 12.6 MHz. It was observed that the FWHM of the saturated absorption spectrum was significantly greater than the natural linewidth of strontium atoms (7.4 kHz). This is attributed to the fact that the strontium atoms in the system are heated to 610 °C, causing the linewidth to be greatly influenced by factors such as interatomic collisions, ultimately broadening it to the order of MHz. Additionally, it was noticed that the scanned strontium atomic spectrum contained many sharp peaks, primarily because the used 689 nm laser was without any frequency stabilization.

## 5. Conclusions

By comparing the cavity mode sizes under cold-cavity conditions and high-power pumping, the equivalent thermal focal length of the Ti:S crystal during the generation of a high-power 689 nm laser was calculated, enabling a quantitative analysis of the thermal characteristics of the Ti:S crystal operating at the gain edge. Based on this, a coupling system capable of achieving optimal mode matching under high-power conditions and a high-efficiency resonator designed for the 689 nm wavelength band were developed. Benefiting from this specialized design, a single-frequency continuous-wave 689 nm laser with an output power of 3.65 W was finally achieved under 15.8 W of a pump laser, and the corresponding optical-to-optical conversion efficiency was 23.1%, which is comparable to that of a Ti:S crystal operating at the gain center. The wavelength tuning range of the obtained 689 nm laser was 65 nm (685 nm–750 nm), with beam quality factors better than 1.1 in both the X and Y directions. The frequency drift was measured as 23.7 MHz/10 s, and the continuous-frequency tuning range reached 17 GHz. Finally, a strontium atomic saturated absorption spectroscopy setup was constructed, and the transition absorption spectrum of strontium atoms near 689 nm was obtained by scanning with the developed continuous single-frequency tunable laser source. Notably, this work demonstrated that the realized single-frequency continuous-wave tunable 689 nm laser holds promising potential as a reliable laser source for quantum computing and quantum simulation systems based on strontium atomic ensembles.

## Data Availability

All data reported in the paper are presented in the main text. Any other data will be provided on request.
